# Preliminary result of definitive radiotherapy in patients with non-small cell lung cancer who have underlying idiopathic pulmonary fibrosis: comparison between X-ray and proton therapy

**DOI:** 10.1186/s13014-019-1221-4

**Published:** 2019-01-28

**Authors:** Hakyoung Kim, Hongryull Pyo, Jae Myoung Noh, Woojin Lee, Byoungsuk Park, Hye Yun Park, Hongseok Yoo

**Affiliations:** 1 Department of Radiation Oncology, Samsung Medical Center, Sungkyunkwan University School of Medicine, 81 Irwon-ro, Gangnam-gu, Seoul, 06351 Republic of Korea; 2 Internal Medicine, Samsung Medical Center, Sungkyunkwan University School of Medicine, Seoul, Republic of Korea

**Keywords:** Idiopathic pulmonary fibrosis, Non-small cell lung cancer, Radiotherapy, Complication, Proton therapy

## Abstract

**Background:**

Idiopathic pulmonary fibrosis (IPF) is associated with fatal complications after radiotherapy (RT) for lung cancer patients; however, the role of proton therapy to reduce the incidence of life-threatening complications is unclear. Herein, we present the preliminary results of early-stage lung cancer patients having IPF and treated with RT, with a focus on the comparison between X-ray and proton therapy.

**Methods:**

From January 2010 to October 2017, we retrospectively reviewed the medical records of 264 patients with stage I-II non-small cell lung cancer (NSCLC) treated with definitive RT alone. Ultimately, 30 patients (11.4%) who had underlying IPF were analyzed. Among these, X-ray and proton RT were delivered to 22 and 8 patients, respectively. Treatment-related complications and survival outcomes were compared between X-ray and proton therapy.

**Results:**

The median follow-up duration was 11 months (range, 2 to 51 months). All living patients were followed-up at least 9 months. Treatment-related death occurred in four patients (18.2%) treated with X-ray but none with proton therapy. Most patients died within one month after the onset of pulmonary symptoms in spite of aggressive treatment. In addition, the 1-year overall survival (OS) rate in patients treated with X-ray and proton was 46.4 and 66.7%, respectively, and patients treated with proton therapy showed a tendency of better survival compared to X-ray (*p* = 0.081). Especially, in GAP stage II and III subgroups, patients treated with proton therapy showed significantly increased survival outcomes compared to X-ray (1-year OS rate; 50.0% versus 26.4%, *p* = 0.036) in univariate analysis.

**Conclusions:**

RT is associated with serious treatment-related complications in patients with IPF. Proton therapy may be helpful to reduce these acute and fatal complications.

**Trial registration:**

retrospectively registered.

## Background

Surgical resection is the standard of care in the treatment of patients with early-stage non-small cell lung cancer (NSCLC). However, a significant number (30–60%) of patients are medically inoperable due to the reasons including; old age, poor performance status, medical co-morbidities, and inadequate cardio-pulmonary function. Patients deemed unsuitable for surgery are often referred for definitive radiation therapy (RT). For the patients with poor pulmonary function or pulmonary diseases such as interstitial lung disease (ILD), even RT can result in significant complications; however, no universally accepted criteria to define patient suitability for RT have been described.

Pulmonary toxicity is one of the most common treatment-related complications after RT, and can affect the morbidity and mortality of patients with lung cancer [1–4]. Severe pulmonary toxicity occurs at a rate of 1.5–20% in patients who undergo stereotactic body radiation therapy (SBRT) or at a rate of 5.0–25% after conventionally fractionated RT [5]. Several studies suggest that idiopathic pulmonary fibrosis (IPF) increases the risk of serious, sometimes life-threatening, treatment-related pulmonary complications such as acute exacerbation of IPF and/or pneumonia following RT [6–8]. Recently, a few clinical results suggest that proton therapy can be performed more safely in patients with IPF than surgery or X-ray treatment [9–11]. However, the role of proton therapy for these vulnerable patients to reduce the incidence of severe RT-related pulmonary complications is still unclear.

Proton therapy for lung cancer patients has started from January 2016 at authors’ institution. Herein, we present the preliminary result of early-stage lung cancer patients who had underlying IPF and were treated with definitive RT, focusing on the comparison between X-ray and proton therapy.

## Methods

### Patients

After Institutional Review Board approval (#2018–08-108), we retrospectively reviewed the medical records of 264 patients with stage I-II NSCLC treated with definitive RT alone at Samsung Medical Center from January 2010 to October 2017. They were determined to be unsuitable for surgery due to various reasons including old age, poor pulmonary function, and poor performance status. All the diagnosis of underlying pulmonary diseases was confirmed by experienced pulmonologists (H.Y.P. and H.S.Y.). Among these, patients who had no underlying pulmonary disease (65 patients, 24.6%) or other than IPF, such as chronic obstructive pulmonary disease (COPD; 152, 57.6%) or combined pulmonary fibrosis and emphysema (CPFE; 17, 6.4%) were excluded. Ultimately, 30 patients (11.4%) that had underlying IPF were analyzed in the current study. The RT technique (X-ray or proton therapy) was selected individually by the discretion of radiation oncologists. Most of all, patients with severely compromised pulmonary function and/or underlying disease, such as COPD, CPFE, and IPF, were mainly treated with proton therapy. And large tumor size and multiple lesions were also considered. In this study, X-ray and proton RT were delivered to 22 (73.3%) and eight (26.7%) patients, respectively. Treatment-related complications and survival outcomes were compared between X-ray and proton therapy.

### Diagnostic and staging scheme for lung cancer and IPF

All tumors were staged based on the seventh edition of the American Joint Committee on Cancer tumor staging criteria. Tumor assessment consisted of complete history taking, physical examination, complete blood counts, chemistry profiles, pulmonary function test (PFT), chest X-ray, computed tomography (CT) scan of the chest and upper abdomen, whole-body ^18^F-fluorodeoxyglucose positron emission tomography with CT (FDG-PET-CT) scan, and magnetic resonance imaging of the brain as a routine staging work-up. The PFT including both spirometry and diffusing capacity were performed before treatment. Detailed measurements included (1) forced expiratory volume in one second (FEV_1_), (2) forced vital capacity (FVC), (3) ratio of the two volumes (FEV_1_/FVC), (4) diffusing capacity of the lung for carbon monoxide (DLCO), and (5) DLCO divided by alveolar ventilation (DLCO/VA). For the diagnosis of IPF, the presence of a typical radiological pattern, which is the coarse reticulation with honeycombing appearance in peripheral and predominantly basal lung area, was seen on high-resolution CT (HRCT). Spirometry typically revealed a reduction in the vital capacity and DLCO. The patients with IPF also showed oxygen desaturation with a six-minute walk test. The GAP model, including four baseline variables (sex, age, and two lung physiology variables; FVC and DLCO) was used for IPF staging [12].

### Radiation treatment

The gross tumor volume (GTV) was delineated under the lung window setting. The internal target volume (ITV) was delineated following four-dimensional CT with consideration of respiratory tumor motion. The clinical target volume (CTV) was generated with a 5 mm expansion of the GTVs in all directions, and was then modified considering the adjacent anatomic structures. The planning target volume (PTV) was generated with 5 mm expansion of the CTVs. The percentage volume of lung receiving ≥20 Gy (V_20_) of the lung was tried to be kept ≤35% and the mean lung dose was ≤20 Gy. Maximum doses to the spinal cord and esophagus were not to exceed 45 Gy and 60 Gy, respectively. The prescription policy was to deliver at least 97% of the prescribed dose to 95% of the CTVs.

Treatment planning for X-ray, Pinnacle treatment planning system, version 9.2 (Royal Phillips Electronics, Miami, FL) was used to calculate the dose distributions, and two different dose-fractionation schedules were used to deliver 60 Gy in either 20 fractions over 4 weeks or 15 fractions over 3 weeks. The biologically equivalent doses at α/β of 10 Gy (BED_10_) were 78 Gy and 84 Gy, respectively. Dose selection depended on the location, size, and geometry of the tumor in relation to the esophagus. If the shortest distance between the CTV margin and the esophagus was ≥1.5 cm, 60 Gy in 15 fractions was preferred to 60 Gy in 20 fractions. For SBRT for small size and peripherally located tumors, 60 Gy in 4 fractions was delivered. Eleven patients were treated with SBRT, 10 with 3-dimensional conformal RT (3DCRT), and one with intensity-modulated RT (IMRT) as it was difficult to safely cover the whole disease extent while satisfying the dose-volume constraints using the 3DCRT technique.

Treatment planning for proton, RayStation treatment planning system, version 6.2 (Raysearch Laboratories AB, Stockholm, Sweden) was used. Line scanning method was used for the proton therapy in all patients with the proton therapy system at our institute (Sumitomo, Japan). Six patients were treated with stereotactic body proton therapy (SBPT, 60–64 CcGE in 4–8 fractions) and two with intensity-modulated proton therapy (IMPT, 60 CcGE in 20 fractions).

### Follow-up

Physical examination, blood tests, chest CT scan, and/or PET-CT were performed every 3 months for 2 years after RT and then every 6 months thereafter to detect disease progression during follow-up. Revised Response Evaluation Criteria In Solid Tumors (RECIST) guidelines (Version 1.1) were used for tumor response evaluation. Treatment-related pulmonary complications, excluding infection-related cases, were evaluated using the Common Terminology Criteria for Adverse Events version 4.0.

### Statistical analysis

Overall survival (OS) was defined as the time from the start date of the RT until the date of death from any cause or the latest documented follow-up. The 1-year rate of OS was calculated using the Kaplan–Meier method and was compared using the log-rank test. Factors that were thought to be relevant were entered into a Cox proportional hazard regression analysis to account for potential confounding factors and to determine independent prognostic factors. To compare the clinical characteristics and dose-volume parameters, Chi-square or Fisher’s exact test was used to assess categorical variables and independent-sample t test was used to assess continuous variables. A *p* value< 0.05 was regarded as statistically significant in two-tailed tests. Statistical analysis was performed using SPSS software, standard version 24.0 (IBM Corporation, Armonk, NY, USA).

## Results

### Patient and tumor characteristics

Overall clinical characteristics according to treatment were described in Table 1. The median age of the population was 76 years (range, 62 to 85 years). Clinical characteristics were similar between the X-ray and proton groups, except for ECOG performance status. Patients who underwent proton therapy showed poorer pulmonary function compared to those undergoing X-ray, although the difference was not statistically significant. When the DLCO were stratified into two categories (> 60% vs. ≤ 60%), the numbers of patients that had poor diffusing capacity in X-ray and proton group were 13/22 (59.1%) and 6/8 (75.0%), respectively. In addition, in the aspect of GAP index, the numbers of patients that presented with high scores (GAP stage II and III) in X-ray and proton groups were 13/22 (59.1%) and 7/8 (87.5%), respectively. In terms of planning dose-volume parameters (Table 2), the CTV was significantly larger in the proton group than in the X-ray group (*p* = 0.007), while the normal tissue doses generally met the constraints and dose-volume parameters for lung, heart, esophagus, and spinal cord were generally similar. Of note, the mean lung dose and the percentage volumes of lung receiving ≥5 Gy and ≥ 20 Gy were similar between the two groups.

**Table 1:** Clinical characteristics according to treatment (*N* = 30)

Characteristics	X-ray (*n* = 22)	Proton (*n* = 8)	*p* value
Age [years; median (range)]	75 (55–84)	77 (62–85)	0.640
Sex			
Female	0 (0%)	1 (12.5%)	0.092
Male	22 (100%)	7 (87.5%)	
Smoking Status			
Never smoker	0 (0%)	1 (12.5%)	0.092
Current or Ex-smoker	22 (100%)	7 (87.5%)	
ECOG performance			
0–1	13 (59.1%)	8 (100%)	0.031
2–3	9 (40.9%)	0 (0%)	
Clinical Stage			
IA-IB	15 (68.2%)	4 (50.0%)	0.361
IIA-IIB	7 (31.8%)	4 (50.0%)	
Histology			
Adenocarcinoma	5 (31.3%)	2 (28.6%)	0.775
Squamous cell carcinoma	10 (62.5%)	5 (71.4%)	
Others	1 (6.2%)	0 (0%)	
Not proven	6	1	
Pulmonary function test			
FEV_1_			
> 50%	21 (95.5%)	8 (100%)	0.540
≤ 50%	1 (4.5%)	0 (0%)	
DLCO			
> 60%	9 (40.9%)	2 (25.0%)	0.424
≤ 60%	13 (59.1%)	6 (75.0%)	
DLCO			
> 40%	20 (90.9%)	7 (87.5%)	0.783
≤ 40%	2 (9.1%)	1 (12.5%)	
GAP index			
1	9 (40.9%)	1 (12.5%)	0.144
2–3	13 (59.1%)	7 (87.5%)	

*ECOG* Eastern Cooperative Oncology Group, *FEV*_*1*_ forced expiratory volume in 1 s; *DLCO*, diffusing capacity of the lung for carbon monoxide

**Table 2:** Dose-volume parameters according to treatment (*N* = 30)

Characteristics	X-ray (*n* = 22)	Proton (*n* = 8)	*p* value
Clinical target volume (cm^3^)	73.3 ± 52.6	206.1 ± 100.6	0.007
Total lung			
Mean dose (cGy or CcGE)	809.8 ± 368.1	796.9 ± 475.2	0.938
V_5_ (%)	28.4 ± 9.7	23.1 ± 11.6	0.216
V_10_ (%)	19.8 ± 8.8	18.7 ± 10.5	0.782
V_20_ (%)	13.1 ± 8.1	14.3 ± 8.6	0.717
Heart			
V_45_ (%)	0.2 ± 0.5	0.1 ± 0.2	0.855
Esophagus			
Maximum dose (cGy or CcGE)	1588.5 ± 1030.2	1261.4 ± 1216.3	0.469
Mean dose (cGy or CcGE)	408.0 ± 301.4	257.9 ± 304.6	0.239
Spinal cord			
Maximum dose (cGy or CcGE)	1141.4 ± 761.5	1487.0 ± 1491.0	0.548

*V*_*D*_ percentage volume of organ receiving ≥ D Gy

Data are presented as mean ± SD

### Survival outcomes and treatment-related complications

**Fig. 1:**
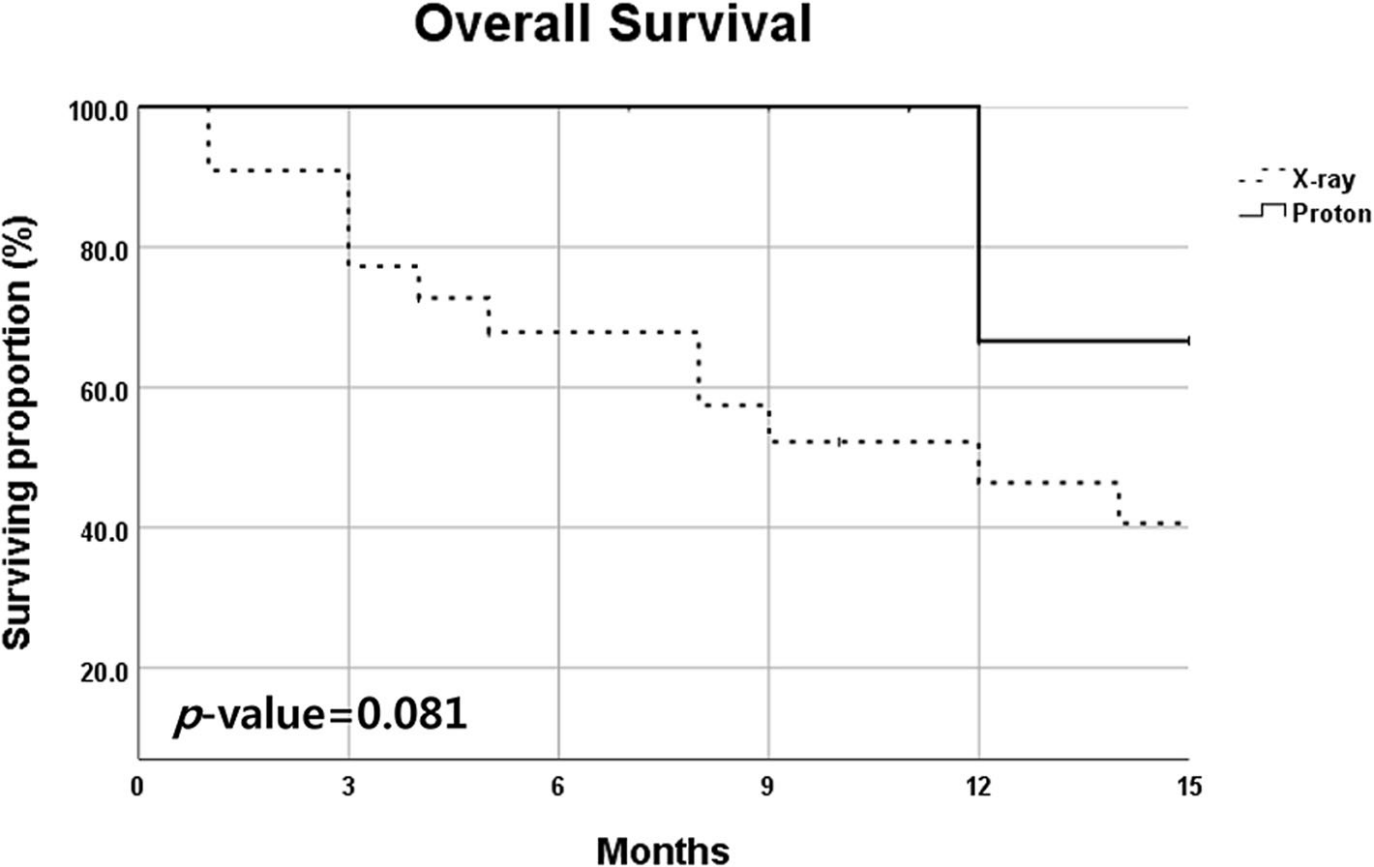
Overall survival curves according to treatment; 1-year OS rate in patients of X-ray and proton groups were 46.4 and 66.7%, respectively

The median follow-up duration was 11 months (range, 2 to 51 months). The 6-month OS rates in patients treated with X-ray and proton were 67.9 and 100%, and the 1-year OS rate was 46.4 and 66.7%, respectively (Table 3). Patients treated with proton therapy showed a tendency of better survival compared to X-ray (*p* = 0.081) (Fig. 1). And only the occurrence of severe pulmonary complication was significantly associated with decreased OS (73.7% vs. 24.0%, *p* = 0.009), while performance status (*p* = 0.986) and RT technique (*p* = 0.318) were not associated with OS in univariate analysis. There was no statistically significant prognostic factors for OS in multivariate analysis after adjusting confounding factors.

**Table 3:** Survival outcomes in patients having IPF (*N* = 30)

Characteristics	X-ray (*n* = 22)	Proton (*n* = 8)	*p* value
*All patients*			
Treatment-related death	4/22 (18.2%)	0/8 (0%)	0.140
6 months OS	67.9%	100%	0.081
1 year OS	46.4%	66.7%	
*GAP stage I*	*n* = 9	*n* = 1	
6 months OS	88.9%	100%	0.501
1 year OS	76.2%	100%	
*GAP stage II-III*	*n* = 13	*n* = 7	0.036
6 months OS	52.7%	100%	
1 year OS	26.4%	50.0%	

*IPF* idiopathic pulmonary fibrosis, *OS* overall survival

**Fig. 2:**
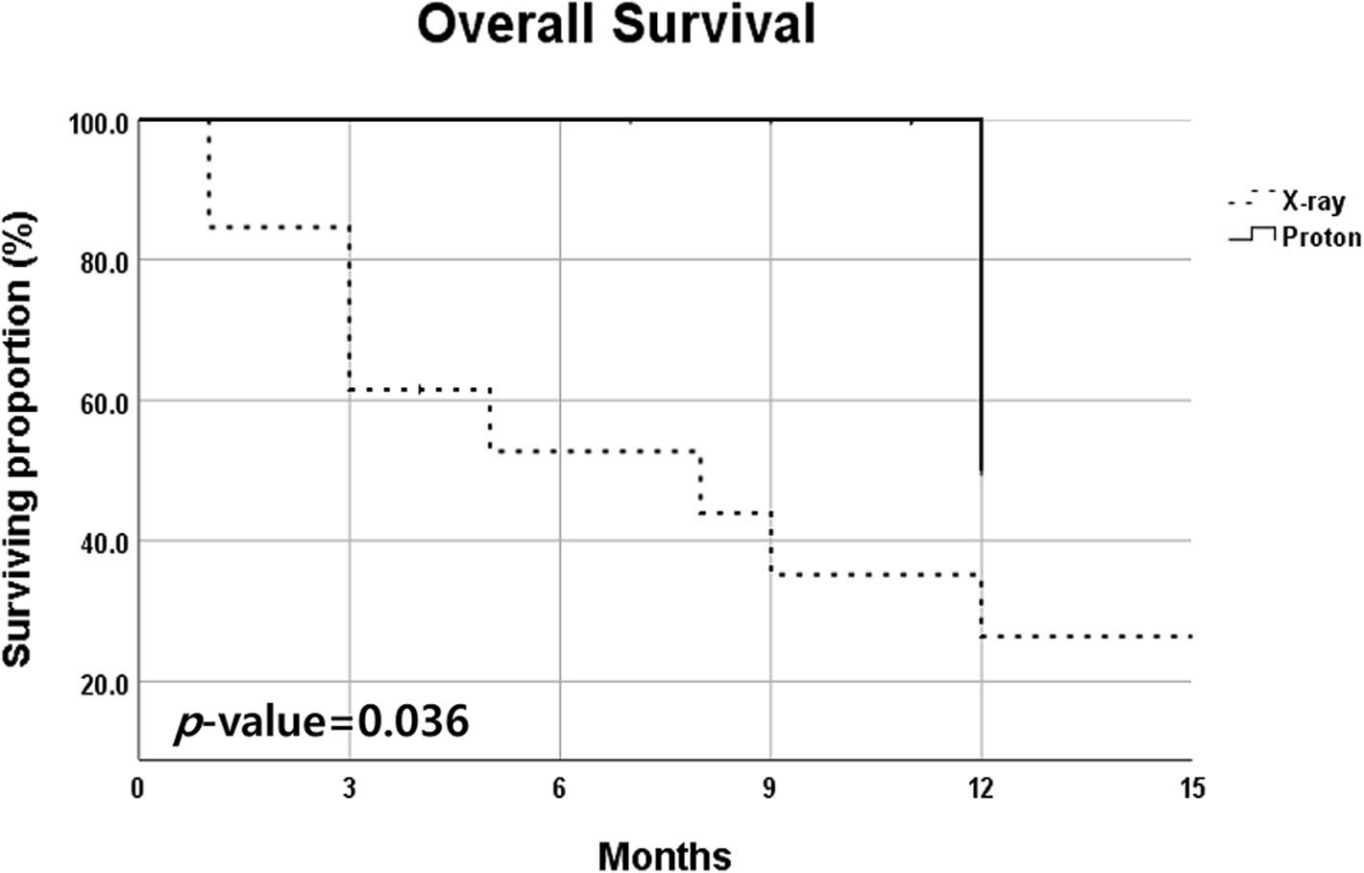
Overall survival curves according to treatment in GAP stage II and III subgroups; 1- year OS rate in patients of X-ray and proton groups were 26.4 and 50.0%, respectively

A statistically significant difference of survival was shown based on GAP stage, and the 1-year OS rate in stage I, II, and III was 78.8, 46.3, and 0%, respectively (*p* = 0.024). In subgroup analysis based on GAP stage, patients treated with proton therapy showed significantly increased survival outcome compared to X-ray in GAP stage II and III groups (1-year OS rate; 50.0% versus 26.4%, *p* = 0.036, Fig. 2). However, there was no statistically significant prognostic factors for OS in multivariate analysis after adjusting confounding factors.

The incidence of severe treatment-related pulmonary complications did not differ significantly between X-ray and proton groups, although it was observed less frequently in the proton group (40.9% vs. 12.5%, *p* = 0.222). All living patients were followed-up at least 9 months, and treatment-related death occurred in four patients (18.2%) treated with X-ray but none with proton therapy (Table 3). Most patients died within one month after the onset of symptom in spite of aggressive treatment. The clinical courses of patients are described in Table 4.

**Table 4:** Clinical courses of patients with fatal treatment-related death

No.	Age/Sex	PS	Smoking	FEV_1_ / DLCO	Underlying disease	Stage	RT technique	Time to TRC (months)	Survival (months)
1	77/M	1	Ex-, 30PY	2.2 L, 84% / 58%	IPF and COPD	T2a N0	3DCRT 60 Gy/15 Fx’s	3	4
2	79/M	2	Ex-, 40PY	2.3 L, 85% / 77%	IPF	T1b N1	3DCRT 60 Gy/15 Fx’s	2	3
3	78/M	2	Ex-, 100PY	2.5 L, 131% / 60%	IPF and COPD	T2a N0	SBRT, 60 Gy/4 Fx’s	2	8
4	69/M	1	Ex-, 120PY	2.0 L, 78% / 31%	IPF	T2a N0	SBRT 60 Gy/4 Fx’s	4	5

*PS* performance status, *PY* pack-year, *FEV*_*1*_ forced expiratory volume in 1 s, *DLCO* diffusing capacity of the lung for carbon monoxide, *IPF* idiopathic pulmonary fibrosis, *COPD* chronic obstructive pulmonary disease, *RT* radiation therapy, *3DCRT* 3-dimensional conformal radiation therapy, *SBRT* stereotactic body radiation therapy, *TRC* treatment-related complications

## Discussion

IPF is a pulmonary disease associated with a dismal prognosis. The median survival of patients with IPF ranges from 2.5 years to 3.5 years and the 5-year survival ranges between 20 and 40% [13]. The condition can be acutely exacerbated by a range of triggering factors, including infection, surgery, and RT. Yamashita et al. [6] demonstrated that severe pulmonary toxicity was observed in nine out of thirteen patients with IPF after SBRT, and seven of these cases were fatal. The presence of IPF shadow on CT was found to correlate well with the severe pulmonary toxicity. However, there was no correlation between the DVH parameters and the severe pulmonary toxicity. This may imply that even a small volume of RT including SBRT is not safe. Accordingly, they suggested that pre-screening for an IPF pattern on CT should be performed before SBRT in patients with lung cancer and underlying IPF. Lee et al. [7] evaluated the relationship between interstitial lung changes in the pre-radiotherapy CT and symptomatic pulmonary toxicity. The risk of pulmonary toxicity grade ≥ 2, ≥3, or ≥ 4 was higher in patients with interstitial lung change than patients without (grade 2, 15.6 to 46.7%, *p* = 0.03; grade 3, 4.4 to 40%, *p* = 0.002; grade 4, 4.4 to 33.3%, *p* = 0.008). Four out of the five patients with grade 5 pulmonary toxicity had diffuse interstitial changes on pre-radiotherapy CT. Recently, Ono et al. [9] reported the clinical results of proton RT in patients with IPF. The cumulative incidence of pulmonary toxicity was 19.8%, including one case of treatment-related death. This study was the first report on the incidence of pulmonary toxicity after proton RT in IPF patients. They showed that grade 4 or 5 pulmonary toxicity occurred at a rate of 6.3%, which was lower than that reported in previous studies with X-ray treatment. This result suggests that proton RT may reduce the incidence of life-threatening pulmonary complications in patients with IPF compared to that with X-ray irradiation.

**Fig. 3:**
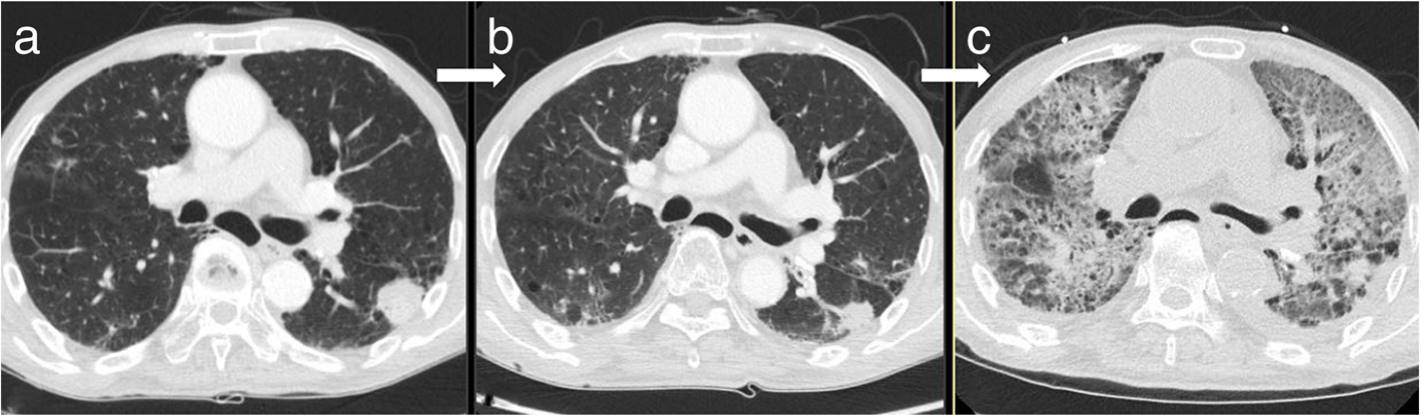
Chest CT axial imaging of the patient who showed grade 5 radiation pneumonitis after radiotherapy; (3a) Pretreatment chest CT image, (3b) At 1 month follow-up, and (3c) At 2 months follow-up

Current study has several limitations. First, it is a retrospective analysis and there may be some selection biases. Second, the sample size is too small to show a statistical significance of difference between the two groups. Despite the lack of statistical significance due to small number of cases, our preliminary analysis is the first one to directly compare X-ray and proton therapy for lung cancer patients with IPF, and shows a promising result that is concordant with previous studies (summarized in Table 5). Although patients that received proton therapy had poorer diffusing capacity and higher GAP stages compared to X-ray, the incidence of severe treatment-related pulmonary complications was observed less frequently in the proton group (40.9% in X-ray vs. 12.5% in proton), and the incidence of treatment-related death occurred in four patients (18.2%) treated with X-ray (Fig. 3) but none with proton therapy. In addition, patients treated with proton therapy showed a tendency of better overall one-year survival compared to X-ray. Especially, proton therapy showed significantly increased survival outcomes compared to X-ray in patients with higher GAP stage (II and III) subgroups (1-year OS rate; 50.0% versus 26.4%, *p* = 0.036). These promising results with proton therapy may be because proton therapy could affect a smaller lung volume than X-ray irradiation. However, there were no statistically significant differences in the dosimetric factors of the patients, including MLD, V_5_, V_10_ and V_20_ of lung although CTV was significantly larger in the proton RT group. We hope our ongoing prospective study with a large number of patients and systematic evaluation of pre- and post-treatment lung functions could confirm the benefit of proton therapy for patients with severely compromised pulmonary function and/or IPF.

**Table 5:** The previous studies reporting severe RP after radiotherapy in patients with IPF

	Number of Patients	Median follow-up (month, range)	Treatment	Median total dose	Grade ≥ 4 RP
Yamashita et al.	13	14.7 (0.3–76.2)	SBRT	48.0 Gy	7 (53.8%)
Lee et al.	14	15.5 (6.1–40.9)	3DCRT	56.9 Gy	5 (35.7%)
Yamaguchi et al.	16	–	SBRT	48.0 Gy	2 (12.5%)
Ono et al.	16	12.0 (4–39)	PBT	80.0 Gy (RBE)	1 (6.3%)
Current study	30	11.0 (2–51)	X-ray^a^/PBT	60.0 Gy	18.2% (4/22) in X-ray 0% (0/8) in PBT

*RP* radiation pneumonitis, *IPF* idiopathic pulmonary fibrosis, *SBRT* stereotactic body radiation therapy, *3DCRT* 3-dimensional conformal radiation therapy, *PBT* proton beam therapy

^a^X-ray treatment includes 3DCRT, SBRT, and intensity-modulated radiation therapy (IMRT)

## Conclusions

Our retrospective analysis showed that RT is associated with serious treatment-related complications in patients with IPF. Proton therapy may be helpful to reduce these acute and fatal pulmonary complications in these vulnerable patients. Currently, a phase 2 prospective trial with proton RT in patients with severely compromised pulmonary function and/or IPF is ongoing at authors’ institution.

## Abbreviations

3DCRT: 3-Dimensional conformal RT
COPD: Chronic obstructive pulmonary disease
CPFE: Combined pulmonary fibrosis and emphysema
CT: Computed tomography
CTV: Clinical target volume
DLCO: Diffusing capacity of the lung for carbon monoxide
DLCO/VA: DLCO divided by alveolar ventilation
FDG-PET-CT: 18F-fluorodeoxyglucose positron emission tomography with CT
FEV_1_: Forced expiratory volume in one second
FVC: Forced vital capacity
GTV: Gross tumor volume
HRCT: High-resolution CT
ILD: Interstitial lung disease
IMRT: Intensity-modulated RT
IPF: Idiopathic pulmonary fibrosis
ITV: internal target volume
NSCLC: Non-small cell lung cancer
OS: Overall survival
PFT: Pulmonary function test
PTV: Planning target volume
RT: Radiotherapy
SBRT: Stereotactic body radiation therapy
V_20_: Percentage volume of lung receiving ≥ 20 Gy
